# Supplementary material for: Unveiling the Bioactive Potential of the Invasive Jellyfish Phyllorhiza punctata Through Integrative Transcriptomic and Proteomic Analyses
Source: Biomolecules. 2025 Aug 4;15(8):1121. doi: 10.3390/biom15081121 (PMC12383608; doi:10.3390/biom15081121)
Supplement: Supplementary file 1 [file biomolecules-15-01121-s001.zip › Suplementary Material/FileS3/PEPFOLD4/XP_065053468_9177/clusters.html]

 PEP-FOLD clusters


Download archive of all models  here   

|  |  |  |
| --- | --- | --- |
| # | file | sOPEP |
| # cluster 1 - Size: 5 |  |  |
|  | XP\_065053468\_9177-model1 | -33.0492 |
|  | XP\_065053468\_9177-model1.1 | -29.309 |
|  | XP\_065053468\_9177-model1.2 | -27.0038 |
|  | XP\_065053468\_9177-model1.3 | -25.6222 |
|  | XP\_065053468\_9177-model1.4 | -25.6054 |
| # cluster 2 - Size: 2 |  |  |
|  | XP\_065053468\_9177-model2 | -29.6101 |
|  | XP\_065053468\_9177-model2.1 | -22.409 |
| # cluster 3 - Size: 2 |  |  |
|  | XP\_065053468\_9177-model3 | -29.1403 |
|  | XP\_065053468\_9177-model3.1 | -28.8766 |
| # cluster 4 - Size: 4 |  |  |
|  | XP\_065053468\_9177-model4 | -28.6348 |
|  | XP\_065053468\_9177-model4.1 | -25.785 |
|  | XP\_065053468\_9177-model4.2 | -23.5285 |
|  | XP\_065053468\_9177-model4.3 | -23.0709 |
| # cluster 5 - Size: 1 |  |  |
|  | XP\_065053468\_9177-model5 | -28.1818 |
| # cluster 6 - Size: 4 |  |  |
|  | XP\_065053468\_9177-model6 | -27.9602 |
|  | XP\_065053468\_9177-model6.1 | -27.0771 |
|  | XP\_065053468\_9177-model6.2 | -23.3568 |
|  | XP\_065053468\_9177-model6.3 | -23.0387 |
| # cluster 7 - Size: 1 |  |  |
|  | XP\_065053468\_9177-model7 | -27.6242 |
| # cluster 8 - Size: 1 |  |  |
|  | XP\_065053468\_9177-model8 | -27.402 |
| # cluster 9 - Size: 4 |  |  |
|  | XP\_065053468\_9177-model9 | -27.2827 |
|  | XP\_065053468\_9177-model9.1 | -24.0016 |
|  | XP\_065053468\_9177-model9.2 | -22.675 |
|  | XP\_065053468\_9177-model9.3 | -17.3335 |
| # cluster 10 - Size: 1 |  |  |
|  | XP\_065053468\_9177-model10 | -26.7538 |
| # cluster 11 - Size: 2 |  |  |
|  | XP\_065053468\_9177-model11 | -26.5781 |
|  | XP\_065053468\_9177-model11.1 | -22.2162 |
| # cluster 12 - Size: 3 |  |  |
|  | XP\_065053468\_9177-model12 | -25.9109 |
|  | XP\_065053468\_9177-model12.1 | -24.3613 |
|  | XP\_065053468\_9177-model12.2 | -22.0585 |
| # cluster 13 - Size: 1 |  |  |
|  | XP\_065053468\_9177-model13 | -25.805 |
| # cluster 14 - Size: 2 |  |  |
|  | XP\_065053468\_9177-model14 | -25.5648 |
|  | XP\_065053468\_9177-model14.1 | -16.3655 |
| # cluster 15 - Size: 1 |  |  |
|  | XP\_065053468\_9177-model15 | -25.383 |
| # cluster 16 - Size: 1 |  |  |
|  | XP\_065053468\_9177-model16 | -25.0502 |
| # cluster 17 - Size: 1 |  |  |
|  | XP\_065053468\_9177-model17 | -24.7864 |
| # cluster 18 - Size: 1 |  |  |
|  | XP\_065053468\_9177-model18 | -23.4122 |
| # cluster 19 - Size: 1 |  |  |
|  | XP\_065053468\_9177-model19 | -23.3999 |
| # cluster 20 - Size: 1 |  |  |
|  | XP\_065053468\_9177-model20 | -23.1919 |
| # cluster 21 - Size: 1 |  |  |
|  | XP\_065053468\_9177-model21 | -23.0407 |
| # cluster 22 - Size: 1 |  |  |
|  | XP\_065053468\_9177-model22 | -22.913 |
| # cluster 23 - Size: 2 |  |  |
|  | XP\_065053468\_9177-model23 | -22.8087 |
|  | XP\_065053468\_9177-model23.1 | -18.634 |
| # cluster 24 - Size: 1 |  |  |
|  | XP\_065053468\_9177-model24 | -22.4336 |
| # cluster 25 - Size: 1 |  |  |
|  | XP\_065053468\_9177-model25 | -22.3422 |
| # cluster 26 - Size: 2 |  |  |
|  | XP\_065053468\_9177-model26 | -21.7657 |
|  | XP\_065053468\_9177-model26.1 | -20.3739 |
| # cluster 27 - Size: 3 |  |  |
|  | XP\_065053468\_9177-model27 | -21.7621 |
|  | XP\_065053468\_9177-model27.1 | -20.3717 |
|  | XP\_065053468\_9177-model27.2 | -19.8778 |
| # cluster 28 - Size: 2 |  |  |
|  | XP\_065053468\_9177-model28 | -21.7188 |
|  | XP\_065053468\_9177-model28.1 | -21.6696 |
| # cluster 29 - Size: 2 |  |  |
|  | XP\_065053468\_9177-model29 | -21.6111 |
|  | XP\_065053468\_9177-model29.1 | -20.2545 |
| # cluster 30 - Size: 1 |  |  |
|  | XP\_065053468\_9177-model30 | -21.5141 |
| # cluster 31 - Size: 1 |  |  |
|  | XP\_065053468\_9177-model31 | -21.4788 |
| # cluster 32 - Size: 3 |  |  |
|  | XP\_065053468\_9177-model32 | -21.3645 |
|  | XP\_065053468\_9177-model32.1 | -19.2335 |
|  | XP\_065053468\_9177-model32.2 | -18.9819 |
| # cluster 33 - Size: 3 |  |  |
|  | XP\_065053468\_9177-model33 | -20.8337 |
|  | XP\_065053468\_9177-model33.1 | -20.1217 |
|  | XP\_065053468\_9177-model33.2 | -19.4524 |
| # cluster 34 - Size: 1 |  |  |
|  | XP\_065053468\_9177-model34 | -20.7193 |
| # cluster 35 - Size: 1 |  |  |
|  | XP\_065053468\_9177-model35 | -20.5169 |
| # cluster 36 - Size: 1 |  |  |
|  | XP\_065053468\_9177-model36 | -20.2981 |
| # cluster 37 - Size: 1 |  |  |
|  | XP\_065053468\_9177-model37 | -20.0785 |
| # cluster 38 - Size: 2 |  |  |
|  | XP\_065053468\_9177-model38 | -20.066 |
|  | XP\_065053468\_9177-model38.1 | -19.5477 |
| # cluster 39 - Size: 1 |  |  |
|  | XP\_065053468\_9177-model39 | -19.8094 |
| # cluster 40 - Size: 1 |  |  |
|  | XP\_065053468\_9177-model40 | -19.7862 |
| # cluster 41 - Size: 1 |  |  |
|  | XP\_065053468\_9177-model41 | -19.6451 |
| # cluster 42 - Size: 1 |  |  |
|  | XP\_065053468\_9177-model42 | -19.6013 |
| # cluster 43 - Size: 1 |  |  |
|  | XP\_065053468\_9177-model43 | -19.5353 |
| # cluster 44 - Size: 1 |  |  |
|  | XP\_065053468\_9177-model44 | -19.4806 |
| # cluster 45 - Size: 1 |  |  |
|  | XP\_065053468\_9177-model45 | -19.3402 |
| # cluster 46 - Size: 1 |  |  |
|  | XP\_065053468\_9177-model46 | -19.3329 |
| # cluster 47 - Size: 2 |  |  |
|  | XP\_065053468\_9177-model47 | -19.1866 |
|  | XP\_065053468\_9177-model47.1 | -16.3036 |
| # cluster 48 - Size: 1 |  |  |
|  | XP\_065053468\_9177-model48 | -19.0613 |
| # cluster 49 - Size: 2 |  |  |
|  | XP\_065053468\_9177-model49 | -18.8946 |
|  | XP\_065053468\_9177-model49.1 | -18.5083 |
| # cluster 50 - Size: 2 |  |  |
|  | XP\_065053468\_9177-model50 | -18.6995 |
|  | XP\_065053468\_9177-model50.1 | -18.5099 |
| # cluster 51 - Size: 1 |  |  |
|  | XP\_065053468\_9177-model51 | -18.2573 |
| # cluster 52 - Size: 1 |  |  |
|  | XP\_065053468\_9177-model52 | -18.03 |
| # cluster 53 - Size: 2 |  |  |
|  | XP\_065053468\_9177-model53 | -17.3997 |
|  | XP\_065053468\_9177-model53.1 | -16.5413 |
| # cluster 54 - Size: 1 |  |  |
|  | XP\_065053468\_9177-model54 | -16.869 |
| # cluster 55 - Size: 1 |  |  |
|  | XP\_065053468\_9177-model55 | -15.9543 |
| # cluster 56 - Size: 2 |  |  |
|  | XP\_065053468\_9177-model56 | -15.5555 |
|  | XP\_065053468\_9177-model56.1 | -14.9979 |
| # cluster 57 - Size: 1 |  |  |
|  | XP\_065053468\_9177-model57 | -15.5413 |
| # cluster 58 - Size: 1 |  |  |
|  | XP\_065053468\_9177-model58 | -15.5342 |
| # cluster 59 - Size: 2 |  |  |
|  | XP\_065053468\_9177-model59 | -15.3481 |
|  | XP\_065053468\_9177-model59.1 | -15.0068 |
| # cluster 60 - Size: 1 |  |  |
|  | XP\_065053468\_9177-model60 | -15.277 |
| # cluster 61 - Size: 1 |  |  |
|  | XP\_065053468\_9177-model61 | -14.8871 |
| # cluster 62 - Size: 1 |  |  |
|  | XP\_065053468\_9177-model62 | -14.6087 |
| # cluster 63 - Size: 1 |  |  |
|  | XP\_065053468\_9177-model63 | -14.4866 |
| # cluster 64 - Size: 1 |  |  |
|  | XP\_065053468\_9177-model64 | -13.8586 |
|  |  |  |
